# Supplementary material for: Coping strategies of COVID-19 recovered patients at the Ghana Infectious Disease Centre
Source: PLoS One. 2025 Jan 8;20(1):e0310921. doi: 10.1371/journal.pone.0310921 (PMC11709276; doi:10.1371/journal.pone.0310921)
Supplement: Attachment — Submitted filename: Response to Reviewers-coping.docx [file pone.0310921.s003.docx]

The Editor

PLOS ONE

Dear Editor,

**Response to Reviewers: Coping Strategies of COVID-19 recovered patients at the Ghana Infectious Disease Centre**

We are grateful to the editor and reviewers for their feedback and helpful recommendations. We are glad to have the chance to resubmit an improved version of our paper for your further evaluation.

In the following, we address the reviewers’ comments and indicate the relevant modifications we applied to the paper. The line numbers we refer to are from the revised manuscript with the track changes off.

**Editor’s comments:**

We noted in your submission details that a portion of your manuscript may have been presented or published elsewhere. “This is the second manuscript in line with our study on quality of life and coping strategies of COVID-19 recovered patients in Ghana. We have submitted revisions to the first paper i.e. quality of life to this same journal and it is under review. However, the analysis conducted here is not a duplication of the previous paper. We only used some of the results or concepts, particularly quality of life to determine the relationship between coping strategies and quality of life.”

Please clarify whether this [conference proceeding or publication] was peer-reviewed and formally published. If this work was previously peer-reviewed and published, in the cover letter please provide the reason that this work does not constitute dual publication and should be included in the current manuscript.

**Response:** This is the first and only submission of this manuscript anywhere, which forms part of a bigger study on Assessment of The Quality of Life and Coping Strategies of COVID-19 Recovered Patients at The Ghana Infectious Disease Centre (GIDC). The content and issues presented for this manuscript is unique to this publication although the study population remains the same. We have included this explanation in the cover letter. Also, the study on quality of life of COVID-19 recovered patients has recently been published in this journal. <https://journals.plos.org/plosone/article?id=10.1371/journal.pone.0306118>

**Reviewer #1:**

1. Post COVID-19 condition, commonly known as long COVID is defined as the continuation or development of new symptoms 3 months after the initial SARS-CoV-2 infection, with these symptoms lasting for at least 2 months with no other explanation (https://www.who.int/europe/news-room/fact-sheets/item/post-covid-19-condition). The authors can explain the reasons the participants were at least one month into the recovery period were eligible.

**Response:** The study aimed to examine patients who had recovered from COVID-19 at the GIDC and were subsequently discharged for treatment at the post-recovery clinic, where eligibility for inclusion in the study was having spent at least one month there. The focus was not exclusively on those with long-COVID, although some of these patients would have been part of the eligible population.

2. The authors need to describe more clearly how to collect 150 participants out of 798 patients, and the process of collecting data.

**Response:** Our study population and sampling strategy has been described in the methods section. Kindly refer to lines 162-169 of the revised manuscript.

3. For the Brief-COPE, EQ-5D-5L, and EQ-VAS questionnaires, how to translate them into local languages and validate.

**Response:** The official language in Ghana is English which is taught from the basic levels of education. The participants in the study all had some form of formal education and so the study tool was well understood by all, which was facilitated by the researchers.

4. The method part was quite long and had unnecessary repetition. Could the author please shorten it?

**Response:** We have read over the methods session and made necessary revisions notifying the repetitions and removing them from the text.

5. The abstract should not exceed 300 words

**Response:** The abstract has been revised to suit the requirements. (Ref to line numbers 21 – 49)

**Reviewer #2:**

1. Great title, well written manuscript from the abstract, introduction, methodology, statistical analysis, results, discussion till conclusion. Thanks for letting me review this great work, Thanks to the authors

**Response:** We thank the reviewer for the feedback.

Thank you
